# Prevention and reversal of selenite-induced cataracts by N-acetylcysteine amide in Wistar rats

**DOI:** 10.1186/s12886-017-0443-1

**Published:** 2017-04-26

**Authors:** Yasaswi Maddirala, Shakila Tobwala, Humeyra Karacal, Nuran Ercal

**Affiliations:** 1 Department of Chemistry, Missouri University of Science and Technology, Rolla, MO 65409 USA; 2 Department of Ophthalmology, Washington University, St. Louis, MO 63110 USA

**Keywords:** Cataract, N-acetylcysteine amide (NACA), Glutathione, Calpain, Crystallin, Lens, Sodium selenite

## Abstract

**Background:**

The present study sought to evaluate the efficacy of N-acetylcysteine amide (NACA) eye drops in reversing the cataract formation induced by sodium selenite in male Wistar rat pups.

**Methods:**

Forty male Wistar rat pups were randomly divided into a control group, an N-acetylcysteine amide-only group, a sodium selenite-induced cataract group, and a NACA-treated sodium selenite-induced cataract group. Sodium selenite was injected intraperitoneally on postpartum day 10, whereas N-acetylcysteine amide was injected intraperitoneally on postpartum days 9, 11, and 13 in the respective groups. Cataracts were evaluated at the end of week 2 (postpartum day 14) when the rat pups opened their eyes. N-acetylcysteine amide eye drops were administered beginning on week 3 until the end of week 4 (postpartum days 15 to 30), and the rats were sacrificed at the end of week 4. Lenses were isolated and examined for oxidative stress parameters such as glutathione, lipid peroxidation, and calcium levels along with the glutathione reductase and thioltransferase enzyme activities. Casein zymography and Western blot of m-calpain were performed using the water soluble fraction of lens proteins.

**Results:**

Morphological examination of the lenses in the NACA-treated group indicated that NACA was able to reverse the cataract grade. In addition, glutathione level, thioltransferase activity, m-calpain activity, and m-calpain level (as assessed by Western blot) were all significantly higher in the NACA-treated group than in the sodium selenite-induced cataract group. Furthermore, sodium selenite- injected rat pups had significantly higher levels of malondialdehyde, glutathione reductase enzyme activity, and calcium levels, which were reduced to control levels upon treatment with NACA.

**Conclusions:**

The data suggest that NACA has the potential to significantly improve vision and decrease the burden of cataract-related loss of function. Prevention and reversal of cataract formation could have a global impact. Development of pharmacological agents like NACA may eventually prevent cataract formation in high-risk populations and may prevent progression of early-stage cataracts. This brings a paradigm shift from expensive surgical treatment of cataracts to relatively inexpensive prevention of vision loss.

## Background

Cataract is a progressive loss of transparency of the lens that affects vision. It is the most common cause of curable blindness, accounting for more than 50% of cases worldwide [1]. Most cataracts are age-related and, according to the National Eye Institute (NEI), by age 80, more than half of all Americans either have a cataract or have had cataract surgery. The NEI and Prevent Blindness America estimate that more than 22 million people in the U.S. live with cataracts and, by 2020, this may exceed 30 million. Although cataract is not associated with signs such as pain or redness in the eye, it is still characterized by symptoms such as blurry vision, double vision, glare of light, and fading of colors. Presently, the only effective treatment option is the surgical removal of the affected lens and replacement with an artificial lens. However, the cost of surgery and need for highly trained personnel to perform ocular surgery limit restoration of vision in certain parts of the world. Unfortunately, the majority of people with vision impairment due to cataracts live in developing countries where access to surgery is limited [2]. Hence, there is an immediate necessity for prevention and non-invasive treatment of cataracts.

The pathogenesis of cataracts is multifactorial, involving mechanisms that are not fully understood, with oxidative stress being the foremost cause in initiating cataract formation [3–9]. Oxidative stress implies an imbalance between the rate of oxidant production and the rate of detoxification, with the rate of production being significantly higher compared to that of detoxification by antioxidants [10]. An organism counteracts this condition with its natural antioxidant defense systems, but with age, oxidants accumulate while antioxidant defenses gradually diminish, which is likely the most important mechanism in age-related cataract formation. Furthermore, Shearer [11] proposed the involvement of oxidative stress in selenite-induced cataract and the sequence of events leading to cataract formation. It was hypothesized that the oxidative damage caused by selenite possibly involves oxidation of critical sulfhydryl groups on calcium ATPase or ion channels. [11] This may lead to altered metabolism in lens epithelium and loss of calcium ATPase activity and, hence, an accumulation of calcium in the nucleus of the lens, which could activate m-calpain. Activation of m-calpain initiates the proteolysis of β-crystallins, leading to the insolubilization of both proteolyzed β- and α-crystallins, co-precipitation of γ-crystallins, and ultimately the formation of cataracts. Since oxidative stress is implicated in cataract formation, application of an antioxidant capable of successfully penetrating the lens tissue represents a logical approach to counteract oxidation-related cataractogenesis. Several compounds with antioxidant properties have been reported to prevent selenite-induced cataracts, such as resveratrol [12], saffron [13], ellagic acid [14], garlic [15], melatonin [16], drevogenin D [17], caffeine [18], ebselen [19], lycopene in vivo [20], N-acetyl-L-carnitine in vitro and in vivo [21–23], and *Ocimum sanctum* in vivo and ex vivo [24]. In fact, N-acetylcysteine (NAC), a glutathione (GSH) prodrug, has previously been tried in selenite-induced cataracts in vivo [25] and was shown to prevent oxidative damage to the lens, slowing down cataractogenesis.

Use of antioxidants in treating or preventing oxidative stress-related disorders is becoming more popular, with thiol antioxidants, such as GSH, cysteine, and NAC, showing some protection [26–28]. However, the negative charge on the NAC molecule at physiological pH renders it less able to permeate cell membranes. This results in the use of higher doses and extended treatment schedules. In contrast, N-acetylcysteine amide (NACA), an analog of NAC, has been shown to be more effective than NAC, owing to its neutral amide group, which increases its lipophilicity and, consequently, its penetration into cell membranes. This has been verified in previous studies, as well as its ability to chelate Cu^2+^, scavenge free-radicals, and protect against oxidative stress [29–36]. Our lab has demonstrated NACA’s improved antioxidant ability compared to that of NAC [33, 37]. Furthermore, NACA is effective at lower concentrations than NAC, which helps to prevent the various adverse effects that are associated with administration of higher doses of NAC [26, 31, 38–40].

Based on these results, the primary objective of this study was to assess the ability of NACA to prevent and/or reverse selenite-induced cataract formation in Wistar rats. We demonstrated that NACA, when injected intraperitoneally on postpartum days 9, 11, and 13 helped to prevent dense nuclear cataract formation [41, 42]. Continuous NACA administration (from postpartum day 15 to 30) as an eye drop formulation reversed the density of the cataract. Our results demonstrate that NACA administration bolstered antioxidant defenses of selenite-dosed animals by replenishing GSH, inhibiting lipid peroxidation, preventing accumulation of calcium, thereby preventing activation of m-calpain and eventually preserving the lens crystallin proteins. Therefore, NACA shows promise as a candidate for development into a drug-based cataract treatment.

## Methods

### Materials

Sodium selenite (Na_2_SeO_3_, Cat no: 214,485, ≥99% pure) and N-(1-pyrenyl) maleimide (NPM, Cat no: P7908, ≥98.5% pure) were purchased from Sigma-Aldrich (St. Louis, MO, USA). N-acetylcysteine amide (NACA, ≥99.9% pure) was provided by Dr. Glenn Goldstein (David Pharmaceuticals, New York, NY, USA). Casein zymogram gels (12% Ready Gel, Cat no: 161–1168) were purchased from Bio-Rad (Hercules, CA, USA). Rabbit m-calpain Antibody (Cat no: 2539S), GAPDH Rabbit mAb (Cat no: 2118S), and Anti-rabbit IgG, HRP-linked Antibody (Cat no: 7074S) were purchased from Cell Signaling Technology (Beverly, MA, USA). All other chemicals were purchased from Sigma-Aldrich, unless stated otherwise.

### Animal study design

Lactating female Wistar rats with 40 2-day-old male pups were purchased from the breeding facility at Charles River and were housed in a temperature- and humidity-controlled (~22 °C and ~55%) animal facility, with a 12-h light and dark cycle. The animals had unlimited access to rodent chow and water and were utilized after a week of acclimation. All animal procedures conformed to the ARVO Statement for the Use of Animals in Ophthalmic and Vision Research and by the Animal Care and Use Protocol Review Committee at the Missouri University of Science and Technology. The rats were divided into four groups, (1) control, (2) NACA, (3) Na_2_SeO_3_, and (4) NACA + Na_2_SeO_3_, such that each group had one lactating female rat with 10 male pups. All rat pups in the NACA and NACA + Na_2_SeO_3_ groups received an intraperitoneal injection of NACA (250 mg/kg bodyweight) once a day on postpartum days 9, 11, and 13 to investigate the preventive effect of NACA on selenite-induced cataracts, whereas the control and Na_2_SeO_3_ groups received an intraperitoneal injection of phosphate buffer (pH 7.4). On postpartum day 10, all rat pups in the Na_2_SeO_3_ and NACA + Na_2_SeO_3_ groups received an intraperitoneal injection of sodium selenite (19 μmol/kg body weight), whereas the control and NACA groups received equivalent volumes of phosphate buffer (pH 7.4). One percent NACA eye drops, prepared in phosphate buffer (pH 7.4), were started from postpartum day 15 (the day that the rat pups opened their eyes) and continued through postpartum day 30 to check for the reversal effect of NACA on the grade of cataracts in the NACA + Na_2_SeO_3_ group (Table 1). Cataracts were graded after examination with a slit lamp microscope on postpartum day 15 before starting the eye drops and on the last day (postpartum day 30) before sacrificing the pups. All rats were anesthetized 2–3 h after administration of the last NACA or buffer eye drop by intraperitoneal injection of ketamine (80 mg/kg) and xylazine (15 mg/kg). The mass of each rat pup was measured at the beginning and the end of the study. Lenses were removed immediately after euthanasia, and promptly placed on dry ice. Samples were stored at a temperature of −80 °C for further analysis.

**Table 1:** Treatment groups studied

Experimental Groups (*n* = 10)	Injections on P9,11, and 13	Injection on P10	Eye Drops
Control	Buffer	Buffer	Buffer
NACA group	NACA (250 mg/kg)	Buffer	1% NACA
Na_2_SeO_3_ group	Buffer	Na_2_SeO_3_(19 μmol/kg)	Buffer
NACA + Na_2_SeO_3_group	NACA (250 mg/kg)	Na_2_SeO_3_(19 μmol/kg)	1% NACA

### Morphological examination of rat eyes

Morphological examination of rat eyes was performed similarly to the procedure described in Carey et al. [30]. One hour prior to examination, a drop of both 2.5% phenylephrine hydrochloride and 1% tropicamide ophthalmic solutions were instilled in each eye to initiate mydriasis. Examination was performed with a slit lamp microscope at 10× magnification. Representative photographs were taken of each lens for assessment by a certified ophthalmologist. Cataracts were graded according to the following scale: clear lens, grade 0; lens with slight opacity, grade 1; lens with partial nuclear opacity, grade 2; lens with dense nuclear opacity, grade 3.

### Determination of GSH levels

GSH levels in lens tissues were measured according to an HPLC method developed within our laboratory and described previously [30, 43]. Briefly, each lens was homogenized in a serine borate buffer (pH 7.8), followed by centrifugation at 5000 × g for 10 min at 4 °C. The resulting supernatant was removed and diluted. A 50 μL aliquot of this diluted supernatant was then added to 200 μL of nanopure water. To each sample, 750 μL of 1 mM NPM in acetonitrile was added. The derivatization reaction was allowed to proceed at room for 5 min, after which time 10 μL of 2 N HCl was added to quench the reaction. Each sample was transferred to an HPLC vial through a 0.45 μm pore filter. HPLC separation and analysis were performed on a Finnigan Surveyor system (Thermo Scientific), which included an Auto Sampler Plus, LC Pump Plus, FL plus Detector, and a 250 × 4.6 mm Reliasil ODS-1 C_18_ column with 5 μm packing material (Orochem Technologies Inc., Naperville, IL, USA). This system was used for all subsequent HPLC analyses described herein. The mobile phase was composed of acetonitrile and nanopure water (70:30 *v*/v ACN: H_2_O) with 1 mL/L acetic acid and 1 mL/L phosphoric acid added to the water to adjust the pH to 2.5. An isocratic method was used, with a flow rate of 1 mL/min. Excitation and emission wavelengths for NPM derivatives were set at 330 and 376 nm, respectively.

### Determination of total glutathione and glutathione disulfide (GSSG) levels

Total glutathione content was determined by reverse phase HPLC, and lens homogenate was prepared and diluted as described above for GSH analysis. 50 μL of diluted supernatant was then added to 60 μL of NADPH (2 mg/mL) in nanopure water. To each sample, 20 μL of glutathione reductase (1 unit/mL) was added, followed by 5 min of incubation at room temperature. After incubation, 120 μL of nanopure water was added, and then 750 μL of 1.0 mM NPM was added to derivatize. After 5 min at room temperature, the reaction was quenched by the addition of 10 μL of 2 N HCl. Filtration and injection were performed as described for HPLC analysis of GSH. Data from the original GSH levels and the total GSH levels in each sample were subsequently used to calculate the levels of GSSG present in each sample.

### Determination of malondialdehyde (MDA)

MDA determination was performed following the method described by Draper [44] and used in our previous cataract studies [30]. First, 350 μL of the lens homogenate was added to 550 μL of 5% TCA and 100 μL of 500 ppm butylated hydroxytoluene in methanol. Samples were heated in a boiling water bath for 30 min, cooled in an ice water bath, and then centrifuged. Equal portions of the supernatant and a saturated solution of thiobarbituric acid were mixed, then heated and cooled in the same manner. 500 μL of each sample mixture was then added to 1 mL of n-butanol, vortexed for 5 min, and then centrifuged. The organic phase was passed through a 0.45 μm filter and injected into the HPLC system described above. However, the mobile phase for MDA analysis consisted of 69.4% sodium phosphate buffer, 30% acetonitrile, and 0.6% tetrahydrofuran (by volume). Excitation and emission wavelengths were set to 515 nm and 550 nm, respectively.

### Determination of glutathione reductase (GR) activity

Glutathione reductase (GR) catalyzes the NADPH-dependent reduction of GSSG to GSH. A commercially available kit (OxisResearch, Portland, OR, USA) was used to perform a spectrophotometric assay that monitors the decrease in absorbance at 340 nm resulting from the oxidation of NADPH to NADP^+^. The rate of decrease in absorbance is directly proportional to the GR activity in the samples, which were prepared according to the manufacturer’s instructions.

### Determination of thioltransferase (Ttase) activity

The thioltransferase activity (Ttase) was determined by the method of Wang [45]. Briefly, 100 μL of lens homogenate was added to 800 μL of solution with 0.1 M phosphate buffer (pH 7.5), 0.2 mM NADPH, 0.5 mM GSH, and 0.4 units of glutathione reductase. Each sample was centrifuged, followed by addition of 100 μL of 20 mM hydroxyethyl disulfide. Absorbance at 340 nm was measured for 2 min. Blanks were prepared without hydroxyethyl disulfide.

### Determination of calcium concentration in the lens

The lenses from all groups were analyzed to determine the Ca^2+^ concentration, as described by Elanchezhian [21]. Briefly, lenses were placed in acid-washed, tared glass vials and heated at 100 °C for 20 h, after which time the dry weights were recorded. Concentrated HCl (0.2 mL) was added to digest the lenses overnight and diluted to 1 mL with deionized water. The samples were then centrifuged at 10,000 × g for 10 min to remove insoluble materials. The supernatant fractions were collected to measure the calcium concentration using an atomic absorption spectrophotometer (model Spectra AA-3100, Perkin Elmer), operated with a slit width of 0.5 nm and wavelength set at 422.7 nm. Standards were prepared from CaCO_3_ and deionized water.

### Casein zymography

Casein zymography was performed using the method of Raser [46]. Casein zymogram gels (12% polyacrylamide) were purchased from Bio-Rad. Protein concentration was determined using the Bio-Rad DC Protein Assay with bovine serum albumin as a standard. Zymogram sample buffer was added to an amount of supernatant containing 50 μg of protein, so that final volume was 30 μL. The casein gels were prerun at 100 V for 15 min, 4 °C, with a running buffer containing 25 mM Tris-HCl, 0.05% MCE, 192 mM glycine, and 1 mM EDTA (pH 8.3) before samples were loaded into the wells. 25 μl of each sample were loaded and electrophoresed at 100 V (constant) for 2 h at 4 °C in a running buffer. The gels were then incubated in a calcium buffer (20 mM Tris-HCl, 10 mM DTT, 2 mM calcium, pH 7.4) overnight at room temperature with slow shaking. The gels were then stained by Coomassie Brilliant Blue and destained; achromatic bands of caseinolysis appeared white against a stained background.

### Preparation of water –soluble lens proteins

Four de-capsulated lenses from each group were homogenized in 600 μL of lysis buffer (20 mM Tris-HCl (pH 7.5), 150 mM NaCl, 1 mM Na_2_EDTA, 1 mM EGTA, 1% Triton, 2.5 mM sodium pyrophosphate, 1 mM β-glycerophosphate, 1 mM Na_3_VO_4_, 1 μg/mL leupeptin, and 2 mM PMSF; Cell Signaling Technology, Inc., Danvers, MA, USA) using a glass Dounce homogenizer. The homogenates were centrifuged at 10,000 × g for 15 min at 4 °C, and the recovered supernatants were designated as the water-soluble fraction.

### Western blot of m-calpain

The water-soluble fraction of lens proteins was used to determine m-calpain levels by Western blot as described by Tobwala et al. [47]. Briefly, 75 μg of soluble lens proteins were resolved by electrophoresis on 10% Mini-Protean TGX gels (100 V, 2 h) in a running buffer containing 25 mM Tris-HCl, 192 mM glycine, 0.05% MCE, and 0.1% SDS (pH 8.3). The samples were transferred to PVDF membranes by an iBlot® Gel Transfer Device (Life Technologies, Grand Island, NY, USA), followed by the addition of a blocking reagent (blok-CH Chemiluminescent Blocker, Millipore, Billerica, MA, USA). Membranes were immunoblotted by using the SNAP i.d. 2.0 Protein Detection System (Millipore) with primary antibodies for m-calpain and GAPDH in 1:750 and 1:1000 dilutions respectively. Subsequently, the membrane was incubated in the respective secondary antibody (1:1000) for 10 min at room temperature. Final visualization was carried out with the enhanced chemiluminescence kit (Cell Signaling Technology, Inc., Danvers, MA, USA). The intensity of protein bands was determined by normalizing against the intensity of GAPDH band. The relative band intensity ratio of the treated group over the control group was calculated.

### Determination of protein

The Bradford method was used to quantify protein levels in the lens homogenates [48], except for casein zymography and the Western blot, where the Bio-Rad DC protein assay was used. This was because the lysis buffer (mentioned in the “Preparation of water-soluble lens proteins” section) contained 1% detergent which would interfere with the results of the Bradford assay. Bovine serum albumin was used as the protein standard.

### Statistical analysis

All reported values were represented as the mean ± S.E. of quadruplicates. Statistical analysis was performed using GraphPad Prism software (GraphPad, San Diego, CA, USA). Statistical significance was ascertained by one-way analysis of variance, followed by Tukey’s multiple comparison tests. Values of *p* < 0.05 were considered significant.

## Results

### Effects of Na_2_SeO_3_ and NACA on cataract formation in the lens

**Fig. 1:**
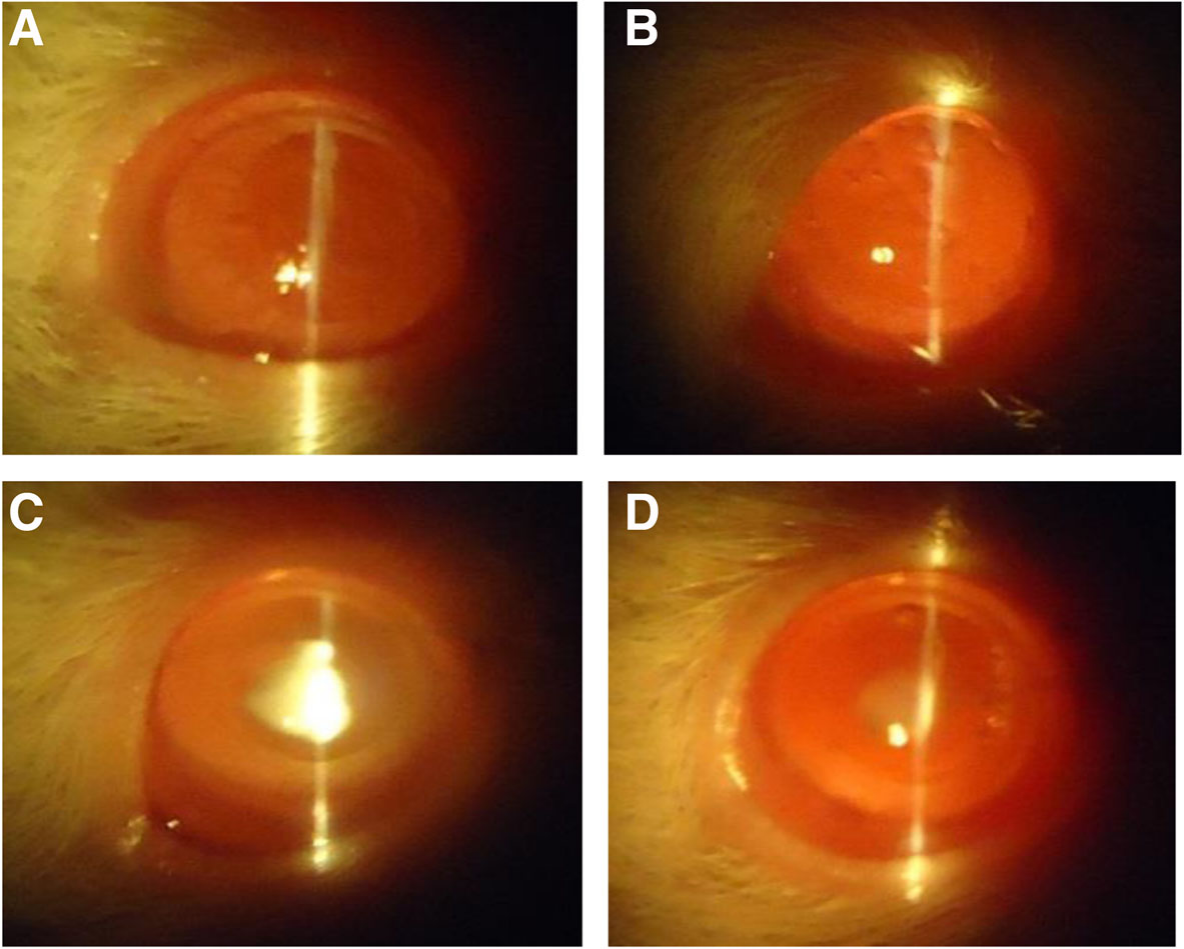
Slit lamp images of eyes. These pictures were taken at week 4 (1 day before sacrifice). A representative picture of the lenses observed for each group is shown. **a**: Lenses from control group were found to be clear with no detectable cataracts; **b**: Lenses from NACA group exhibited similar results as that of control, with no detectable cataracts; **c**: Lenses from Na_2_SeO_3_ group exhibited dense nuclear cataracts (80%); **d**: Lenses from NACA + Na_2_SeO_3_ group exhibited lower grade of cataracts

Intraperitoneal injection of Na_2_SeO_3_ (19 μmol/kg) on postpartum day 10 was sufficient to induce cataract formation, which was visible by the time the rat pups opened their eyes. Inspection of the rat pups’ eyes with a slit lamp microscope confirmed that all animals injected only with Na_2_SeO_3_ developed cataracts: two out of ten (20%) developed grade 2 cataracts and the remaining eight out of ten (80%) developed grade 3 cataracts. In comparison, NACA intraperitoneal injections decreased the severity of cataract formation; eight rats out of ten (80%) developed grade 2 cataracts, while one rat formed grade 1 cataracts, and one rat did not develop any cataracts (grade 0). These results indicated that NACA was successful in preventing cataract formation. By the end of week 4, there was no change in the grades of the cataracts in the Na_2_SeO_3_-injected rat pups (eight out of ten (80%) rats had grade 3 cataracts, and two rats had grade 2 cataracts). However, treatment with NACA eye drops decreased the severity of cataract formation, where five rats out of the ten (50%) had grade 2 cataracts, four rats had grade 1 cataracts, and one rat had no cataract (grade 0). The grading of the lens in all of the groups is tabulated in Table 2, and the slit-lamp pictures of representative lenticular opacities observed for each group are shown in Fig. 1.

**Table 2:** Classification of the degree of cataract formation in rat pups at weeks 2 and 4

Experimental Groups (*n* = 10)	Grade of Cataracts (Week 2)					Grade of Cataracts (Week 4)				
	0	1	2	3		0	1	2	3	
Sham-treated control group	10	0	0	0		10	0	0	0	
NACA group	10	0	0	0		10	0	0	0	
Na_2_SeO_3_ group	0	0	2	8		0	0	2	8	
NACA + Na_2_SeO_3_group	1	1	8	0		1	4	5	0	

Morphological examination of rat lenses at week 2 and week 4. Dense cataract formation was prevented by NACA injection in the NACA + Na_2_SeO_3_ group at the end of week 2 when the rats opened their eyes. Continuation of NACA eye drops until the end of week 4 decreased the number of rats with grade 2 cataracts, reversing them to grade 1.

### Effects of NACA on GSH and GSSG levels in the lens

**Fig. 2:**
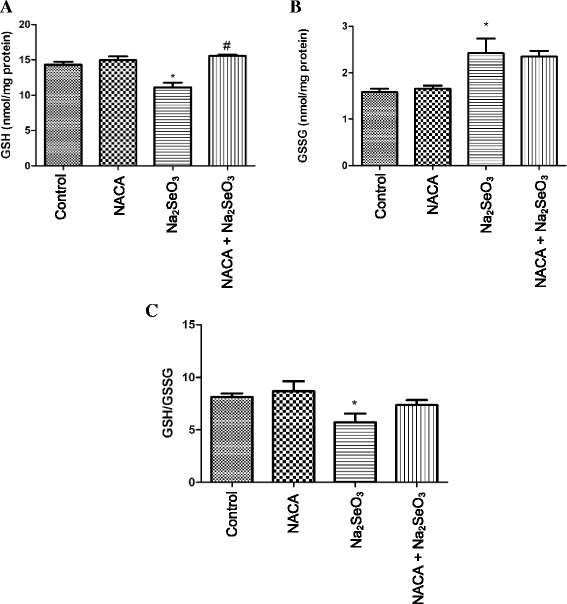
Effect of NACA on (A) GSH, (B) GSSG, and (C) GSH/GSSG in lenses of rat pups injected with Na_2_SeO_3_. **a** Treatment with NACA increased the GSH levels significantly when compared with the Na_2_SeO_3_ group. **b** Treatment with NACA didn’t show a significant decrease in GSSG levels. **c** Treatment with NACA increased the GSH/GSSG ratio, but is neither significantly different from control nor Na_2_SeO_3_ group lenses. Statistical Analysis was performed by one-way analysis of variance, followed by Tukey’s multiple comparison tests. *p < 0.05 compared to the control group and #*p* < 0.05 compared to the Na_2_SeO_3_ group. Values are reported as mean (*n* = 4) ± S.E

GSH levels in lenses from the Na_2_SeO_3_ group were found to be significantly (*p* < 0.05) lower than those of the lenses from the control and NACA groups. However, treatment with NACA in the NACA + Na_2_SeO_3_ group (Fig. 2a) significantly (*p* < 0.05) increased GSH levels compared to the Na_2_SeO_3_ group.

GSSG levels in the lenses from the Na_2_SeO_3_ group were found to be significantly (*p* < 0.05) higher than those in the lenses from the control group. However, NACA treatment (NACA + Na_2_SeO_3_ group) was not able to reduce GSSG levels back to the control (Fig. 2b).

The ratio of GSH and GSSG in the lenses from the Na_2_SeO_3_ group was found to be significantly lower (*p* < 0.05) than in the lenses from the control group, whereas the GSH/GSSG ratio in the NACA treatment group (NACA + Na_2_SeO_3_) was close to the control group lenses (Fig. 2c).

### Effects of NACA on MDA levels

**Fig. 3:**
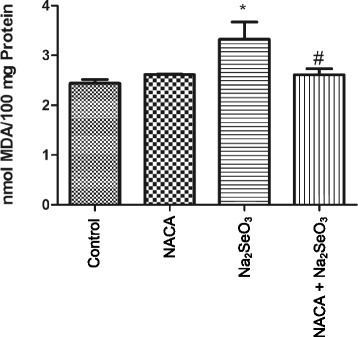
Effect of NACA on MDA levels in rat pups injected with Na_2_SeO_3_. Lenses from sodium selenite injected rat pups showed significantly higher levels of MDA compared to the control, whereas treatment with NACA significantly decreased the MDA levels. Statistical Analysis was performed by one-way analysis of variance, followed by Tukey’s multiple comparison tests. *p < 0.05 compared to the control group and #*p* < 0.05 compared to the Na_2_SeO_3_ group. Values are reported as mean (*n* = 4) ± S.E

MDA levels in the lenses from the Na_2_SeO_3_ group were found to be significantly (*p* < 0.05) higher than those in the control group lenses. However, MDA levels in NACA-treated animals were significantly reduced (*p* < 0.05) and were similar to those in the control group (Fig. 3).

### Effects of NACA on GR and Ttase activities

The GR activity in lenses from the Na_2_SeO_3_ group was found to be significantly (*p* < 0.05) higher than that of the control, NACA, and NACA + Na_2_SeO_3_ groups.

The Ttase activity in the lenses of the Na_2_SeO_3_ group was found to be significantly (*p* < 0.05) lower than that of the control group lenses. However, Ttase activity in the NACA treatment group (NACA + Na_2_SeO_3_) was close to that of the control group (Table 3).

**Table 3:** Activities of antioxidant enzymes in rat pups

Groups (*n* = 4)	Glutathione reductase (GR mU/mg of protein)	Thioltransferase (Ttase mU/mg of protein)
Control	0.77 ± 0.16	2.0 ± 0.28
NACA	0.92 ± 0.063	1.8 ± 0.20
Na_2_SeO_3_	1.3 ± 0.23^*^	1.2 ± 0.19^*^
NACA + Na_2_SeO_3_	0.88 ± 0.16^#^	1.8 ± 0.35

Activities of glutathione reductase and thioltransferase from the lenses of each group. NACA treatment in the NACA + Na_2_SeO_3_ group significantly decreased the glutathione reductase activity when compared to the glutathione reductase activity of Na_2_SeO_3_ group lenses. Although NACA increased the thioltransferase activity in NACA + Na_2_SeO_3_ group, it was not significantly higher when compared to the Na_2_SeO_3_ group. Statistical analysis was performed by one-way analysis of variance, followed by Tukey’s multiple comparison tests. *p < 0.05 compared to the control group and ^#^ *p* < 0.05 compared to the Na_2_SeO_3_ group. Values are reported as mean (*n* = 4) ± S.E.

### Effects of Na_2_SeO_3_ and NACA on calcium levels in the lens

**Fig. 4:**
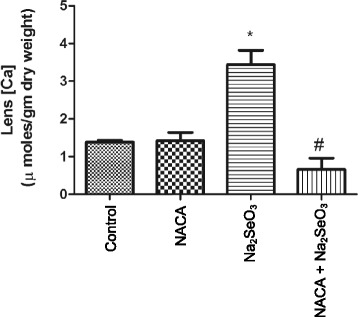
Effect of NACA on calcium levels in rat pups injected with Na_2_SeO_3_ *.* Lens from sodium selenite injected rat pups showed a significantly higher calcium levels compared to the control, whereas treatment with NACA significantly decreased the calcium levels. Statistical Analysis was performed by one-way analysis of variance, followed by Tukey’s multiple comparison tests. *p < 0.05 compared to the control group and #*p* < 0.05 compared to the Na_2_SeO_3_ group. Values are reported as mean (*n* = 4) ± S.E

Lenses from rat pups in the Na_2_SeO_3_ group showed significantly (*p* < 0.05) higher levels of calcium than the lenses in the control and NACA-only groups. However, treatment with NACA showed a significant (*p* < 0.05) reduction in the levels of calcium compared to those of the Na_2_SeO_3_ group, with the levels becoming nearly the same as those of the control (Fig. 4).

### Effects of Na_2_SeO_3_ and NACA on casein zymography

**Fig. 5:**
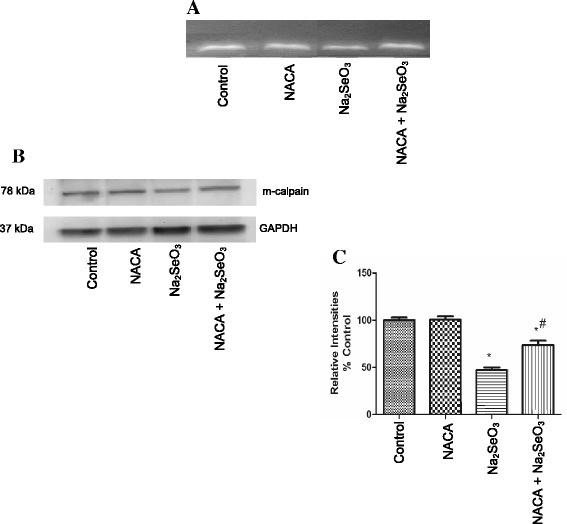
Effect of NACA on **a** Casein zymography and **b** Western blot, and **c** Relative densitometry intensities of m-calpain. **a** Lens from sodium selenite injected rat pups showed a decreased band intensity of m-calpain indicating decreased m-calpain activity compared to the control. However, treatment with NACA preserved the m-calpain activity; **b** Western blot from sodium selenite injected rat pups showed a band intensity of m-calpain compared to control; **c** Western blot results normalized to the levels of GAPDH gene. The graphs represent relative densitometric analysis of treated group lenses over the control group lenses. Statistical Analysis was performed by one-way analysis of variance, followed by Tukey’s multiple comparison tests. *p < 0.05 compared to the control group and #*p* < 0.05 compared to the Na_2_SeO_3_ group. The graph is representative of triplicate gels and the values are reported as mean ± S.E

Elevated calcium levels in Na_2_SeO_3_-treated animals prompted the comparison of m-calpain activation. Casein zymography was performed, wherein calcium-activated calpain-mediated lysis of casein on the gel was measured. The casein zymogram showed a less intense band of m-calpain in the lenses of the Na_2_SeO_3_ group. However, the intensity of the band in the NACA-treated group was close to that of the control group (Fig. 5a).

### Effects of Na_2_SeO_3_ and NACA on the Western blot of m-calpain

To support our results of casein zymography, Western blot analysis of m-calpain protein was performed in the soluble protein fractions of lenses. A less intense band of m-calpain protein was observed in the lenses of the Na_2_SeO_3_ group compared to the control group lenses. However, lenses in the NACA + Na_2_SeO_3_ group showed a more intense band when compared with the Na_2_SeO_3_ group (Fig. 5b, c).

## Discussion

Cataract is the leading cause of blindness worldwide, and surgical replacement of the opacified lens with an artificial lens is currently the only way to remedy vision loss. Although cataract surgery is considered to be very successful in terms of visual outcome, the cost, need for trained personnel, and postsurgical complications limit the worldwide availability and accessibility to this procedure. Hence, development of alternatives to surgical intervention is warranted [49].

Oxidative stress is an imbalance between the rate of oxidant production and degradation [50]. Substantial supporting evidence suggests that reactive oxygen species (ROS) and oxidative damage are involved in the development of cataracts [51, 52]. Cataract formation has a multifactorial etiology. Oxidative stress, resulting in the depletion of antioxidant defense systems in the lens, is considered to be a major factor in the formation of cataracts. Lens transparency is dependent on the preservation of a favorable redox balance, which is, in part, maintained by its high GSH content [53–55]. Decreased levels of GSH in the lens can lead to free radical accumulation, resulting in lipid peroxidation and decreased antioxidant enzyme activity [56, 57], all of which lead to cataract development. Therefore, an alternative method to prevent or treat cataracts would be the use of a thiol antioxidant. Based on this, we have investigated the effects of a novel thiol antioxidant, NACA, in selenite-induced cataracts. Results from morphological observation indicate that NACA was able to prevent and reverse the formation of cataracts in the NACA + Na_2_SeO_3_ group (Table 2).

As discussed earlier, GSH is the most important antioxidant in the lens; it is the first line of defense against oxidative stress [58]. Our results show a significant decrease in GSH levels in the lenses of the Na_2_SeO_3_ group (Fig. 2a) when compared to those of the control group indicating oxidative stress. In addition to a decrease in GSH, increase in intracellular GSSG (Fig. 2b) levels were observed in the lenses of the Na_2_SeO_3_ group. In addition, the GSH/GSSG ratio, an indicator of the overall antioxidative capacity of a cell, was decreased in the lenses of the Na_2_SeO_3_ group. Treatment with NACA significantly increased the GSH levels in the NACA-treated group. This suggests that NACA was able to prevent oxidative stress by restoring GSH levels. This is likely achieved through several processes: supplying cysteine for GSH biosynthesis, reducing extracellular cystine to cysteine [59], and converting GSSG to GSH by non-enzymatic thiol disulfide exchange [31]. Our results are in agreement with previous studies that reported a decrease in GSH levels upon Na_2_SeO_3_ treatment in animal models [14, 19, 60–64].

However, significant improvement in the GSH/GSSG ratio was not observed in the NACA + Na_2_SeO_3_ group (Fig. 2c). Furthermore, changes in the levels of GSH and GSSG were seen to affect the activity of GR. This enzyme regenerates GSH from its oxidized form and is imperative to GSH homeostasis. Increased activity of GR in the lenses of the Na_2_SeO_3_ group could be attributed to the activation of the lens antioxidant defense network against a change in the redox status. However, this increase in activity of GR was not sufficient to convert all of the GSSG to GSH and resulted in a high level of GSSG leading to a lower GSH/GSSG ratio. Furthermore, NACA treatment increased the levels of GSH and restored GR activity (Table 3).

Lipid peroxidation has been associated with cataract formation [65–67]. Polyunsaturated fatty acids are susceptible to free radical attack, initiating a chain reaction that terminates in the formation of stable by-products, such as malondialdehyde (MDA), which are used as markers of lipid peroxidation. Lenses from the Na_2_SeO_3_ group had increased levels of MDA (Fig. 3) when compared to control group lenses, indicating increased lipid peroxidation in the Na_2_SeO_3_ group. This observation is consistent with other studies that reported increased lipid peroxidation upon treatment with Na_2_SeO_3_ [13, 14, 19, 23, 30, 60, 61]. NACA was able to decrease the lipid peroxidation induced by Na_2_SeO_3_ by providing adequate amounts of GSH, which is a substrate for the glutathione peroxidase-catalyzed reduction of hydroperoxides.

A key GSH-dependent enzyme, Ttase, catalyzes the conversion of protein disulfides back to protein thiols using GSH as one of the substrates. During cataract formation, oxidative stress builds up due to diminished levels of GSH and leads to protein-thiol mixed disulfide formation. Crystallin-thiol mixed disulfides then precipitates within the lens causing the cataract [68]. We speculate this may be due to decreased Ttase activity as a consequence of lowered GSH levels during cataract development. Depletion of Ttase activity in cataract formation has been previously reported [45, 69, 70]. Rat pups injected with Na_2_SeO_3_ showed decreased Ttase enzyme activity, as compared to control, leading to the precipitation of crystallin proteins, whereas treatment with NACA restored the Ttase enzyme activity to a level close to that of the control (Table 3).

Precipitation of proteins can also result from calcium uptake in the lens. High concentrations of calcium in the lens nucleus of selenite cataracts have been previously reported by Hightower [71] which may be due to inhibition of Ca-ATPase activity [72]. Accumulation of calcium in the nucleus of the lens leads to m-calpain activation, proteolysis, insolubilization, and precipitation of crystallins. We have observed a 2.5-fold increase in calcium levels in the lenses of the Na_2_SeO_3_ group, as compared to the levels in the control. Our results are in agreement with other studies which previously reported elevated calcium levels in the lenses of selenite-induced cataracts [21, 62, 71, 73]. Treatment of selenite-challenged rats with NACA (NACA + Na_2_SeO_3_ group) prevented such an increase, and maintained the lens calcium levels close to those of control (Fig. 4).

Calpains are cysteine proteases whose activity depends on calcium concentrations. Based on the calcium levels required to activate these calpains, two predominant forms of calpains have been reported, μ-calpain (micromolar concentrations of calcium) and m-calpain (millimolar concentrations of calcium) [74]. Increased calcium levels lead to dissociation of m-calpain subunits, resulting in activation of m-calpain, which hydrolyzes the lens substrates such as crystallins leading to cataractogenesis. However, activation of m-calpain is followed by degradative autolysis of m-calpains [17]. In the present study, casein zymography was performed to check m-calpain activation. Contrary to our expectations based on increased calcium levels, lenses from the Na_2_SeO_3_ group showed decreased m-calpain activity with a less intense band (Fig. 5a). These results could be attributed to the degradative autolysis of m-calpains subsequent to their activation. These results were further supported by performing Western blots of m-calpain. A similar trend with decreased m-calpain levels were observed in the Na_2_SeO_3_ group (Fig. 5b, c). Decreased m-calpain activity and levels can thus be attributed to degradative autolysis following activation of m-calpain [21, 71, 75, 76].

Several preliminary studies were done using this model with different time periods and using NACA eye drops alone. However, we were limited due to this being an acute model of cataract formation. In addition, the time-sensitive and laborious nature of these experiments resulted in the inclusion of fewer animals per treatment group, being a further impediment.

## Conclusion

In summary, our data indicate that oxidative stress plays a role in cataract formation, particularly in glutathione maintenance. For this reason, a GSH prodrug would be effective as a therapeutic agent for prevention and reversal of cataracts. The data support our hypothesis that NACA protects by increasing GSH, reducing MDA levels, restoring enzyme activities, and normalizing calcium levels. Our future studies will focus on investigating the effectiveness of NACA eye drops and its pharmacokinetic profile in different animal models of cataract formation.

Our present and future studies may eventually help prevent cataract formation in high-risk populations and treat early-stage cataracts without need for surgical intervention. This would decrease the social and economic burden of blindness worldwide.

## Abbreviations

ARVO: Association for Research in Vision and Ophthalmology
DTT: Dithiothreitol
GR: Glutathione reductase
GSH: Glutathione
GSSG: Glutathione disulfide
MCE: 2-mercaptoethanol
MDA: Malondialdehyde
NAC: N-acetylcysteine
NACA: N-acetylcysteine amide
NEI: National Eye Institute
PMSF: Phenylmethylsulfonylfluoride
TCA: Trichloroacetic acid
Ttase: Thioltransferase
